# Association between pain and cognitive and daily functional impairment in older institutional residents: a cross-sectional study

**DOI:** 10.1186/s12877-023-04337-8

**Published:** 2023-11-18

**Authors:** Sheng-Hua Wu, Chung-Fen Lin, I-Cheng Lu, Ming-Sung Yeh, Chin-Cheng Hsu, Yuan-Han Yang

**Affiliations:** 1 Department of Anesthesiology, Kaohsiung Municipal Ta-Tung Hospital, Kaohsiung, Taiwan; 2 Department of Anesthesiology, Kaohsiung Medical University Hospital, Kaohsiung, Taiwan; 3 Department of Anesthesiology, School of Medicine, College of Medicine, Kaohsiung Medical University, Kaohsiung, Taiwan; 4 Institute of Population Health Sciences, National Health Research Institutes, 35 Keyan Road, Zhunan, Miaoli County 35053 Taiwan; 5 Department of Anesthesiology, Kaohsiung Municipal Siaogang Hospital, Kaohsiung, Kaohsiung, Taiwan; 6 Department of Family Medicine, Min-Sheng General Hospital, Taoyuan, Taiwan; 7 Department of Health Services Administration, China Medical University, Taichung, Taiwan; 8 Center for Geriatrics and Welfare Research, National Health Research Institutes, Yunlin, Taiwan; 9 Department of Neurology, Kaohsiung Medical University Hospital, Kaohsiung Medical University, Kaohsiung, Taiwan; 10 Neuroscience Research Center, Kaohsiung Medical University, Kaohsiung, Taiwan; 11 Department of Neurology, Kaohsiung Municipal Ta-Tung Hospital, No. 68, Jhonghua 3Rd Road, Cianjin District, Kaohsiung, 80145 Taiwan; 12 Present Address: Post Baccalaureat Medicine, Kaohsiung Medical University, Kaohsiung, Taiwan

**Keywords:** Pain, Dementia, Daily functional dependence, Institutional residents

## Abstract

**Background:**

Pain is often neglected in disabled older population, especially in Taiwan where the population of institutional residents is rapidly growing. Our study aimed to investigate pain prevalence and associated factors among institutional residents to improve pain assessment and management.

**Methods:**

This nationwide study recruited 5,746 institutional residents in Taiwan between July 2019 and February 2020. Patient self-report was considered the most valid and reliable indicator of pain. A 5-point verbal rating scale was used to measure pain intensity, with a score ranging from 2 to 5 indicating the presence of pain. Associated factors with pain, including comorbidities, functional dependence, and quality of life, were also assessed.

**Results:**

The mean age of the residents was 77.1 ± 13.4 years, with 63.1% of them aged over 75 years. Overall, 40.3% of the residents reported pain, of whom 51.2% had moderate to severe pain. Pain was more common in residents with comorbidities and significantly impacted emotions and behavior problems, and the mean EQ5D score, which is a measure of health-related quality of life (*p* < .001). Interestingly, pain was only related to instrumental activities of daily living (IADL) and not activities of daily living (ADL). On the other hand, dementia was significantly negatively associated with pain (*p* < .001), with an estimated odds of 0.63 times (95% CI: 0.53–0.75) for the presence of pain when compared to residents who did not have dementia.

**Conclusions:**

Unmanaged pain is common among institutional residents and is associated with comorbidities, IADL, emotional/behavioral problems, and health-related quality of life. Older residents may have lower odds of reporting pain due to difficulty communicating their pain, even through the use of a simple 5-point verbal rating scale. Therefore, more attention and effort should be directed towards improving pain evaluation in this vulnerable population .

**Supplementary Information:**

The online version contains supplementary material available at 10.1186/s12877-023-04337-8.

## Background

Pain is a subjective experience that involves an unpleasant sensory and emotional response, usually associated with actual or potential tissue damage [1]. As people age, they are more likely to develop various comorbidities, and increase the risk of experiencing pain compared to individuals belonging to other age groups [2]. As the global population ages, there is a high prevalence of pain in the elderly, as reported in previous studies [3–5]. Unfortunately, pain in geriatric individuals is often neglected, particularly in those with functional declines and dementia. We know that people with persistent pain may worsen comorbidities and eventually lead to neurophysiological disorders, such as depression, anxiety, and substance abuse disorders.

In recent years, there has been an increasing number of adults over 65 years living in institutions [6]. The National Study of Long-Term Cares Providers (2013–2014) indicated that approximately 1.4 million people in the United States live in nursing homes [7]. In addition, as of 2018, Taiwan has become an aging society, with over 3.3 million adults over 65 years of age, which is more than 14% of the population [8]. There is a rapidly growing need for care in the aging population, with at least over 250,000 people receiving long-term care services in 2019. Identifying painful conditions in frail institutional residents and conducting comprehensive assessments of potential factors associated with pain, including limited functional abilities that may occur during aging, are crucial to target specific domains.

Previous research has revealed that estimated pain experiences in older adults within institutions ranged from 45 to 80% [9]. Institutional residents, who frequently have multiple comorbidities, are particularly prone to daily pain [10], and more than 20% of these residents receive inadequate or no analgesic treatment [6, 11]. In addition to aging, chronic illnesses are associated with a greater incidence of pain. A review study reported that patients with neuropathological disorders have a high prevalence of pain [12]. Some patients with stroke also showed increased neuronal hyperexcitability and complaints of post-stroke pain [13].

Hoffmann et al.found more than 50% of the 4,584 nursing home residents in Germany had dementia based on the health insurance claims data [14]. Similarly, a recently published nationwide survey in Taiwan showed that the institutional prevalence of dementia was 87.1%, including very mild dementia [15]. An increased in documented dementia cases has been reported especially in institutions in Taiwan [16] and the estimated global prevalence was forecasted to triple by 2050 [17]. The future outcome of demented patients in Asia has been reported to be highly associated with cerebrovascular diseases [18], which can further deteriorate cognition [19]. Accurate evaluating pain in older residents is challenging, as they often have varying degrees of impaired cognitive and communication functions [20, 21].

Additionally, pain in institutional residents may be associated with a decline in their daily function and subsequent disability. Chronic pain has been shown to reduce activities of daily living (ADL) and physical activity in community-dwelling older people [22]. A nationwide cross-sectional study conducted in Spain reported that more than 50% of adults with chronic pain experience some limitations in ADL [23]. A population-based survey indicated that higher age and pain intensity significantly affected ADL [24].

Pain is a significant problem among institutional residents globally, and more research is needed to improve their care, particularly in countries like Taiwan where the population of older adults is rapidly growing. Therefore, our study aims to investigate the prevalence of pain and its associated factors among institutional older adults in Taiwan, with a larger sample size, providing further insights into the relationship between pain and comorbidities, cognition, daily functional dependence, and quality of life. This research is essential to improve care for disabled older adults in Taiwan and other countries facing a similar demographic shift.

## Methods

This cross-sectional study utilized data from the Taiwan National Health Research Institutes and was conducted between July 2019 and February 2020. The study population included 5,746 institutional residents from 22 counties and cities in Taiwan. A total of 299 long-term care institutions were recorded, including 164 welfare institutions for elderly people, 125 general nursing homes, and 10 veteran homes. The study was approved by the Institutional Review Board of the Research Ethics Committee of National Health Research Institutes (EC1080502), and written informed consent was obtained from the residents before their participation. If self-determination was not possible, proxy consent was requested from their families. All methods were conducted according to the Helsinki guidelines and regulations. The response rate was 87.7%, and socio-demographic parameters, pain experiences, current comorbidities (within 6 months), emotional status, and physical functional disabilities were obtained from residents through questionnaires (self- or other-report). In addition, all interviewers or specialists who participated in the study underwent a training course to reduce inter-interviewers’ bias in the cognitive dysfunction and dementia assessment.

The primary outcome of this study was pain score, which was measured by assessing the intensity of pain using a verbal rating scale. The scale consisted of five-points of assessment that corresponded to the following level of pain: 1 (no pain), 2 (slight pain), 3 (moderate pain), 4 (severe pain), and 5 (unbearable pain). This 5-point rating scale is a reliable and valid tool for assessing pain in older adults, both with and without cognitive impairment [25–29]. Pain scores that ranged from 2 to 5 indicated the presence of pain, while scores of 0 to 1 indicated the absence of pain [30]. The study also collected socio-demographic variables, including age (≤ 65, 66–75, > 75 years), sex, and educational level (illiteracy, ≤ 6 years, and > 6 years).

We conducted further analyses to examine the presence of cognitive dysfunction and the prevalence of diagnosed dementia among institutional residents. The Mini-Mental State Examination (MMSE) was commonly used as a brief screening tool for cognitive disorders [31], with lower scores indicating more severe cognitive problems. However, the optimal cutoff values for the MMSE are inconclusive. We used the MMSE questionnaire in the Taiwan/Mandarin version published by Psychological Assessment Resources and compared different cutoff points with the existence of pain. First, we defined a cutoff-score of 27 (26 or below) for the MMSE as indicative of cognitive impairment in highly educated individuals based on previous research [32]. Participants who scored below 27 were further categorized into three levels: mild (20 to 26 points), moderate (10 to 19 points), and severe (0 to 9 points). Second, as individuals with lower levels of formal education may have reduced cognitive performance on the MMSE [33–35], the MMSE cut-off values were adjusted to minimize the effects of educational level. For literate participants, a score below 25 on the MMSE indicated cognitive impairment, while for illiterate participants, the. threshold was set at a score below 14. Additionally, institutional residents who had a diagnosed dementia certificate from a hospital or by a neurologist were surveyed. The severity of dementia was also investigated using the Clinical Dementia Rating Scale (CDR 0 to 3 scores), which is a reliable tool for evaluating cognitive performance across six domains [36]. To classify the severity of dementia, five levels of dementia were defined as follows: 0 (normal), 0.5 (very mild dementia), 1 (mild dementia), 2 (moderate dementia), and 3 (severe dementia).

A painful condition often impairs the moods, functional dependence, and health-related quality of life of individuals. The presence of pain was investigated in relation to several factors including activities of daily living (ADL), instrumental activities of daily living (IADL), emotional or behavioral problems, and the EQ-5D-5L questionnaire as measured by the EQ-5D-5L questionnaire. ADL was scored from 0 to 100, with higher scores indicating better functioning in basic self-care tasks [37]. IADL was scored from 0 to 8, with higher scores indicating independence in performing instrumental activities of daily life [38]. The EQ-5D-5L questionnaire consists of five dimensions: mobility, self-care, activities, pain/discomfort, and anxiety/depression [39]. A higher score indicates better quality of life, with scores ranging between -1.0259 (the worst health status) and 1 (full health) [40]. The covariates of chronic illness in the residents were also examined.

Institutional residents in the study were divided into two groups based on their experience of pain, and various covariates, such as demographics, comorbidities, cognitive functions, and quality of life (as listed in Tables 1, 2, 3 and 4), were compared between the two groups. Continuous data were expressed as the mean ± standard deviation (SD) and analyzed using Student’s *t*-test, while categorical data were expressed as number (%) and analyzed using the chi-square test. Statistical analyses were performed using SAS 9.4 (SAS Institute, Cary, NC). A total of 30 comparisons were made in this study, and to account for multiple testing, the significance level was adjusted to 0.0017 (0.05/30) using the Bonferroni correction. Therefore, a *p*-value less than 0.0017 was considered statistically significant. The odds ratio (OR) and 95% confidence intervals (CI) were calculated using logistic regression analysis after adjusting for age, sex, and educational level.

**Table 1:** Demographic characteristics of institutional residents

Covariates			Pain		*p* value
	All (*N* = 5746)		No (*N* = 3428)	Yes (*N* = 2318)	
Age (y), mean ± SD		77.1 ± 13.4	77.3 ± 13.2	76.6 ± 13.8	.15
Age, *n* (%)					.17
≤ 65 years			611 (17.8)	446 (19.2)	
> 65 years			2817 (82.2)	1872 (80.8)	
Age, *n* (%)					.52
≤ 75 years			1252 (36.5)	866 (37.4)	
> 75 years			2176 (63.5)	1452 (62.6)	
Gender, *n* (%)					.35
Male			1705 (49.7)	1124 (48.5)	
Female			1723 (50.3)	1194 (51.5)	
Education level, *n* (%)					.18
Illiteracy			1286 (38.7)	828 (36.6)	
Literacy with education ≤ 6 years			1146 (34.5)	830 (36.7)	
Literacy with education > 6 years			890 (26.8)	606 (26.8)	
MMSE scores, mean ± SD		17.0 ± 6.66 (*n* = 2058)	16.9 ± 6.69 (*n* = 1075)	17.1 ± 6.63 (*n* = 983)	.37

The total MMSE score ranges from 0 to 30

Abbreviations: *SD* Standard deviation, *MMSE* Mini–Mental State Examination

**Table 2:** Association between pain and comorbidities among institutional residents

Covariates	Pain		aOR^Ι^ (95% CI)	*p* value
	No	Yes		
	*n* (%)	*n* (%)		
Comorbidities			2.24 (1.46—3.43)^*^	.0002^*^
No	97 (2.8)	28 (1.2)		
Yes	3331 (97.2)	2290 (98.8)		
Disease Lists				
Hypertension	2062 (60.2)	1348 (58.2)	0.93 (0.83—1.04)	.18
Diabetes mellitus	1039 (30.3)	694 (30.0)	0.97 (0.87—1.10)	.66
Bone disorders	219 (6.4)	214 (9.2)	1.53 (1.25—1.87)^*^	< .0001^*^
Vision diseases	111 (3.2)	105 (4.5)	1.36 (1.03—1.80)	.030
Stroke	1053 (30.7)	752 (32.5)	1.07 (0.95—1.20)	.29
Coronary artery disease	470 (13.7)	325 (14.0)	1.04 (0.89—1.21)	.63
Arrhythmia	94 (2.7)	54 (2.3)	0.86 (0.60—1.21)	.38
Cancer	82 (2.4)	81 (3.5)	1.42 (1.03—1.95)	.031
Respiratory disorders	410 (12.0)	314 (13.6)	1.15 (0.98—1.35)	.09
Digestive disorders	496 (14.5)	335 (14.5)	0.98 (0.84—1.14)	.76
Urogenital disorders	447 (13.0)	366 (15.8)	1.31 (1.12—1.53)^*^	.0009^*^
Dementia	819 (23.9)	480 (20.7)	0.86 (0.75—0.98)	.027
Psychiatric problems	484 (14.1)	355 (15.3)	1.07 (0.92—1.24)	.40
Mental retard	66 (1.9)	38 (1.6)	0.84 (0.56—1.27)	.41
Cerebral palsy	20 (0.6)	22 (1.0)	1.70 (0.91—3.18)	.09
Parkinsonism	234 (6.8)	159 (6.9)	1.03 (0.83—1.27)	.80
Spinal cord injury	32 (0.9)	63 (2.7)	2.71 (1.75—4.19)^*^	< .0001^*^
Currently Infectious status	18 (0.5)	23 (1.0)	1.93 (1.04—3.59)	.037
Rare diseases	5 (0.2)	11 (0.5)	3.15 (1.09—9.10)	.034
Refractory epilepsy	111 (3.2)	86 (3.7)	1.13 (0.84—1.52)	.41
Others	442 (12.9)	354 (15.3)	1.22 (1.05—1.43)	.010

Abbreviations: *a*OR Adjusted odds ratio, *CI* Confidence intervals

^Ι^aOR: adjusted for age, sex, and educational level

^*^p < .0017

**Table 3:** Relationship between pain and cognitive impairment and dementia

Covariates	Pain		aOR^Ι^ (95% CI)	*p* value
	No	Yes		
Cognitive function, *n* (%)				
Normal (MMSE scores 27 – 30)	112 (10.4)	95 (9.7)	reference	
Impairment^1^ (MMSE scores 0–26)			0.98 (0.72—1.33)	.89
Mild (20 – 26)	251 (23.4)	262 (26.7)	1.17 (0.84—1.63)	.35
Moderate (10 – 19)	576 (53.6)	495 (50.4)	0.85 (0.62—1.18)	.33
Severe (0 – 9)	136 (12.7)	131 (13.3)	0.90 (0.61—1.33)	.60
Cognitive function, *n* (%)				
Normal	280 (26.1)	303 (30.9)	reference	
Impairment^2^ (Literacy with MMSE < 25 or illiteracy with MMSE < 14)	792 (73.9)	678 (69.1)	0.80 (0.65–0.98)	.029
Dementia, *n* (%)				
No	306 (10.8)	310 (16.1)	reference	
Yes	2523 (89.2)	1622 (84.0)	0.63 (0.53—0.75)^*^	< .0001^*^
Dementia, *n* (%)				
No	306 (10.8)	310 (16.1)	Reference	
Had dementia diagnosis certificate from hospital	726 (25.7)	440 ( 22.8)	0.62 (0.50—0.76) ^*^	< .0001^*^
CDR ≥ 0.5 and MMSE < cutoff point^a^	488 (17.3)	430 (22.3)	0.85 (0.69—1.04)	.12
CDR ≥ 1 but MMSE ≥ cutoff point^a^: must ask a neurologist for reconfirmation	1 (0.04)	2 (0.1)	1.96 (0.18—21.7)	.58
CDR ≥ 1	1308 (46.2)	750 (38.8)	0.56 (0.46—0.67) ^*^	< .0001^*^
Clinical Dementia Rating (CDR), n (%)				
0: No dementia	53 (2.1)	44 (2.7)	reference	
.5: very mild dementia	218 (8.7)	165 (10.2)	0.94 (0.60—1.48)	.79
1: Mild dementia	304 (12.2)	248 (15.3)	1.00 (0.65—1.56)	.99
2: Moderate dementia	429 (17.2)	242 (14.9)	0.72 (0.47—1.12)	.14
3: Severe dementia	1489 (59.7)	921 (56.9)	0.78 (0.52—1.19)	.25

The total MMSE score ranges from 0 to 30. We defined individuals having cognitive impairments as following 1. MMSE score with a cutoff of 27 (26 or below). 2. Literacy with MMSE < 25 or illiteracy with MMSE < 14

Abbreviations: *MMSE* Mini–Mental State Examination, *a* OR Adjusted odds ratio, *CI* Confidence intervals, *CDR* Clinical Dementia Rating

^Ι^aOR: adjusted for age, sex, and educational level

^a^ Cutoff point: Literacy with MMSE < 25 or illiteracy with MMSE < 14

^*^p < .0017

**Table 4:** Association of ADL, IADL, mood, and quality of life with pain

Covariates		Pain		aOR^a^ (95% CI)	*p* value
	All	No	Yes		
ADL^b^, mean ± SD	24.9 ± 31.8	24.8 ± 32.2	25.0 ± 31.1		
ADL^b^, *n* (%)					
No dependence (100)		123 (3.6)	68 (2.9)	reference	
Dependence (0—99)				1.20 (0.89—1.63)	.24
Mild (91 – 99)		72 (2.1)	34 (1.5)	0.80 (0.48—1.33)	.40
Moderate (61- 90)		418 (12.2)	292 (12.6)	1.22 (0.87—1.70)	.24
Severe (21 – 60)		646 (18.8)	481 (20.8)	1.29 (0.94—1.79)	.12
Total (0 – 20)		2169 (63.3)	1443 (62.3)	1.18 (0.87—1.60)	.29
IADL^c^, mean ± SD	0.98 ± 1.62	0.94 ± 1.62	1.04 ± 1.61		
IADL^c^, *n* (%)					
No dependence (8)		33 (1.0)	13 (0.6)	reference	
Dependence (0—7)				1.74 (0.91—3.32)	.09
Mild (6 – 7)		79 (2.3)	50 (2.2)	1.57 (0.75—3.26)	.23
Moderate (3 – 5)		368 (10.7)	301 (13.0)	2.08 (1.07—4.02)	.030
Severe (0 – 2)		2948 (86.0)	1954 (84.3)	1.69 (0.89—3.23)	.11
Emotional/behavioral problems, *n* (%)					
No		2259 (65.9)	1408 (60.7)	reference	
Yes		1169 (34.1)	910 (39.3)	1.23 (1.10—1.38)^*^	.0002^*^
Mean EQ5D scores, mean ± SD (health: 1, -1.0259 ~ 1)	-0.04 ± 0.45 (*n* = 5737)	0.09 ± 0.39 (*n* = 3427)	-0.23 ± 0.48 (*n* = 2310)		< .001^*^

Abbreviations: SD = standard deviation; *a*OR = adjusted odds ratio; CI = confidence intervals

^a^aOR: adjusted for age, sex, and educational level

^b^ADL means activities of daily living, and the total score for ADL ranges from 0 to 100

^c^IADL means instrumental activities of daily living, and the total score for IADL ranges from 0 to 8

^*^p < .0017

## Results

Table 1 presents the demographics and baseline MMSE scores of the study population, along with a comparison of those who had pain and those who did not. The sample included 5,746 institutional residents with a mean age of 77.1 ± 13.4 years, of whom18.4% were aged ≤ 65 years, 18.5%. were aged 66–75 years, and 63.1% were aged > 75 years. Of the residents, 49.2% (*n* = 2,829) were men, 81.6% (*n *= 4,689) were aged > 65 years, and 26.8% (*n *= 1,496) had completed at least junior high school education. The mean MMSE score was 17.0 ± 6.66. Demographic characteristics, including age, sex, educational level, and mean MMSE score, showed no association with the presence of pain. Furthermore, up to 40.3% (*n* = 2,318) of the institutional residents reported different levels of pain, with 1,131 residents reporting slight pain, 553 (9.6%) reporting moderate pain, 282 (4.9%) reporting severe pain, and 352 (6.1%) reporting unbearable pain (data not shown). These findings suggest that undertreated pain is not uncommon among institutional residents.

Table 2 shows the current comorbidities in institutional residents and their association with pain. Nearly all residents (97.8%) had comorbidities. Among the most common comorbidities were hypertension (59.3%), stroke (31.4%), diabetes mellitus (30.2%), and dementia (22.6%). The residents with comorbidities were significantly more likely to experience pain, with an odds ratio of 2.24 (95% CI: 1.46–3.43) after adjusting for potential confounders (age, sex, and educational level). We further analyzed the association between pain and specific comorbidities and found that bone disorders (arthritis, bone fracture, osteoporosis) (OR: 1.53, *p* < 0.0001), urogenital disorders (OR: 1.31, *p* < 0.0009), and spinal cord injury (OR: 2.71,* p* < 0.0001) were significantly associated with pain in institutional residents after adjusting for potential confounders.

Table 3 presents the relationship between self-report pain, cognitive dysfunction, and dementia among institutional residents. There was no significant association between pain and cognitive dysfunction, defined as MMSE ≤ 26 (*p* = 0.89 and OR: 0.98, 95% of CI: 0.72–1.33). After adjusting for educational level (literacy or illiteracy), cognitive impairment defined as MMSE < 25 for literacy and MMSE < 14 for illiteracy was not associated with pain. However, our study found a high prevalence of dementia in older institutional residents, and a significant negatively association was observed between dementia and pain (*p* < 0.001 and OR: 0.63, 95% CI: 0.53–0.75) compared to residents without dementia. The distribution of dementia was determined using four criteria: 1. confirmation from hospital, 2. CDR ≥ 0.5 and MMSE < cutoff point (MMSE < 25 for literacy and MMSE < 14), 3. CDR ≥ 1 but MMSE ≥ cutoff point, 4. CDR ≥ 1. Institutional residents with a hospital certificate of dementia diagnosis and those with CDR scores of 1 or above were less likely to report pain, with a p-value less than 0.001, indicating a statistically significant difference. Additionally, a post-hoc analysis of the data based on CDR scores was also conducted, and the results showed no significant difference in the likelihood of reporting pain across different CDR scores.

Table 4 presents the association of pain with functional dependence, emotional/behavioral problems, and quality of life among institutional residents. The majority of residents who reported pain had severe or total dependence in ADL and IADL. However, the mean ADL score and the severity of ADL were not statistically significant in relation to self-reported pain. In contrast, residents who reported pain had higher mean IADL scores than residents who did not report pain (*p* < 0.001), and the moderate dependent group had an estimated 2.08 times the odds of experiencing pain when compared to residents who had no dependence in IADL. These findings suggest that residents with higher IADL scores reported more pain experience. Additionally, we found a strong association between pain and emotional/behavioral problems (*p* = 0.0002 and OR: 1.23, 95% of CI: 1.10–1.38), and pain had a significantly negative effect on quality of life, using the ED5Q assessment tool (*p* < 0.001).

## Discussion

This study is the first large-scale and nationwide study in Taiwan, to our knowledge, that aim to examine pain prevalence and its effects on institutional older residents. The study enrolled 5,746 residents from various institutions in Taiwan, with over 60% aged over 75 years. The results showed that pain is prevalent among institutional residents, with 40.3% experienced pain, and more than half of them (51.2%) having moderate or more pain. These finding suggest that undermanaged pain is a common problem among institutional residents, which is consistent with the prevalence of pain reported in nursing home in other countries such as the US, Canada and Sweden (ranging from 40 to 80%) [41]. The study also found that pain was commonly associated with comorbidities such as bone disorders, urogenital disorders, and spinal cord injury. However, different degrees of dementia were negatively associated with pain. The study also found that the IADL level, but not ADL, was significantly associated with pain. Pain was found to significantly increase mood- and behavior-related problems, and have a significant negative impact on quality of life.

Atee et al [42] conducted a retrospective cross-sectional analysis and found a high prevalence of pain (54.6%-78.6%) among individuals with dementia. Similarly, a review study reported that approximately 60% to 80% of nursing home resident with dementia experienced pain [43]. In the present study, which included over 80% of residents with varying stages of dementia, those with a hospital diagnosis certificate of dementia and those with CDR scores of 1 or above were significantly less like to report pain using a 5-point verbal rating scale. This may be due to reduced communication abilities in these populations, even with a simple numeric scale. A systematic review study proposed that self-report by the patients is a standard tool for pain assessment and should be the first-line approach whenever possible[44] and self-report measures are applicable even in case of moderate dementia [45]. Considering the limited functional abilities of institutional residents, we used a 5-point verbal rating scale to assess their pain instead of traditional visual analogous scale (VAS), which is more difficult to use in clinical practice [46]. Pain identification remains a challenge in institutions, especially in older residents with dementia and at least one chronic disease. Our results showed that even this simpler pain scale may underestimate pain experience by older individuals with moderate to severe cognitive impairment and those with functional dependence. To effectively assess pain in this vulnerable population, we need to pay more attention on pain assessment using more assessment tools and encourage interdisciplinary collaboration. The UK National Guideline recommends using numerical rating scale or verbal descriptors for elderly individuals with mild to moderate cognitive decline. However, for cases with severe cognitive impairment, PAINAD (Pain in Advanced Dementia) and Doloplus-2 scale are recommended [45]. Youjeong et al. have suggested that further studies are needed to assess pain in older adults, and multidimensional tools may be required for accurate self-reporting of pain [47]. This approach can help uncover undiagnosed pain and improve the overall management of pain in this population.

Functional deficits are common among institutional residents, and the decline in physical function may increase the risk of mortality and adverse health outcomes [48, 49]. In our study, most of the residents showed high dependency in ADL and IADL (mean scores: 24.9 ± 31.8 and 0.98 ± 1.62, respectively). A recent study reported that pain significantly predicted a decline in ADL functioning over a 6-month follow-up in order individuals with limited ability to communicate, as determined using a pain assessment checklist (PACSLAC-D) [50]. A cross-sectional study including community-dwelling older adults showed a positive association between pain and functional impairment in participants with cognitive impairment (OR: 1.74, 95% CI: 1.15,2.62; *p* < 0.01). However, a systematic review of 11 studies revealed controversial results in eight of the studies and showed no or weak association between pain and functional impairment. Therefore, we investigated the relationship between pain report and functional deficits in institutional older residents. We observed that the presence of pain showed a significant association with IADL, but not ADL. Other contributing factors may have affected the association between pain and functioning in our study population, in which most residents had cognitive impairment. Basic ADLs comprise daily fundamental skills, whereas IADLs require a more complex capacity to live independently in a community. Declined performance in IADLs is observed in the early stages of dementia, whereas impairments in ADL are often noted in later stages of dementia[38]. In our study, residents with reported pain had a higher IADL level than those without pain (1.04 ± 1.61 and 0.94 ± 1.62, respectively). The residents who were capable of understanding tasks and questions in the questionnaire and express their pain well were considered as having IADL function.

Pain assessment in institutional residents is a complex task that requires more comprehensive and reliable tools to accurately detect pain in. Previous systemic review studies have highlighted the importance of identifying of barriers and facilitators to pain assessment in improving the quality of care provided for nursing home residents [51, 52]. While standard pain assessment tools are still lacking, other reliable assessment tools have been developed and studied for their application in individuals with dementia [53, 54]. However, a recent meta-analysis has shown that the routine use of a pain tool alone is insufficient to evaluate pain experience by patients with dementia and that a comprehensive pain model involving multidisciplinary health professionals is necessary to improve pain management [55]. Supplementary assessment tools such as observational ratings for pain-related behaviors by observers (body movement, facial expression, and vocalization domain) [41, 56–58] and electronic pain recognition tools have also been suggested to aid in pain assessment [59, 60]. Further research is needed to develop and validate more comprehensive pain assessment tools to improve pain management in this vulnerable population.

The strength of our study lies in its large sample size and highly representative nature. However, this study still has some limitations. A previous study reported that the EQ-5D as a self-report tool is suitable for use in older nursing home residents with cognitive impairment, whereas the validity of the EQ VAS was lower [61]. Although we used a simpler 5-point verbal descriptor pain scale with numbers to examine different pain intensities, this tool may have still not reflected the actual extent of pain experienced by the residents as pain is a complex phenomenon and can be more difficult to evaluate in the older population. We plan to incorporate multidimensional pain assessment tools as the next step in our study, particularly to assess pain among residents with multiple health issues. Furthermore, the characteristics, duration and locations of pain as well as current medications that the residents were taking to alleviate pain were not evaluated in this study. More studies that incorporate the aforementioned aspects are warranted in the future.

## Conclusions

Unmanaged pain is common among the institutional residents in Taiwan. Painful conditions impact on functional dependence (IADL), emotional/behavioral problems and quality of life. Patients with a certain degree of cognitive impairment often underestimate their painful conditions, which may subsequently lead to devastating health consequences. Thus, more attention and focus must be given to the evaluation of the pain conditions in this vulnerable population.

### Supplementary Information

**Additional file 1: Supplemental Table 1. **Association between comorbidities and pain for those with dementia. **Supplemental Table 2.** Association between ADL, IADL, Mood, Quality of Life and pain for those with dementia. **Supplemental Table 3.** Association between comorbidities and pain for those without dementia. **Supplemental Table 4.** Association between ADL, IADL, Mood, Quality of Life and pain for those without dementia

## Data Availability

The datasets generated and/or analyzed during the study are not publicly available due to privacy and ethical restrictions but are available from the corresponding author on reasonable request.

## Abbreviations

ADL: Activities of daily living
MMSE: Mini-Mental State Examination
CDR: Clinical dementia rating scale
IADL: Instrumental activities of daily living
VAS: Visual analogous scale
