# Supplementary material for: Association between pain and cognitive and daily functional impairment in older institutional residents: a cross-sectional study
Source: BMC Geriatr. 2023 Nov 18;23:756. doi: 10.1186/s12877-023-04337-8 (PMC10657596; doi:10.1186/s12877-023-04337-8)
Supplement: Supplementary file 1 — Additional file 1: Supplemental Table 1. Association between comorbidities and pain for those with dementia. Supplemental Table 2. Association between ADL, IADL, Mood, Quality of Life and pain for those with dementia. Supplemental Table 3. Association between comorbidities and pain for those without dementia. Supplemental Table 4. Association between ADL, IADL, Mood, Quality of Life and pain for those without dementia [file 12877_2023_4337_MOESM1_ESM.docx]

Supplemental Table 1 Association between comorbidities and pain for those with dementia

| Covariates |  | Pain | | *p* value | Crude OR  (95% CI) | aOR^a^  (95% CI) |
| --- | --- | --- | --- | --- | --- | --- |
|  | All  (N = 4,145) | No  (N = 2,523) | Yes  (N = 1,622) |  |  |  |
|  |  | *n* (%) | *n* (%) |  |  |  |
| Comorbidities |  |  |  | .002^*^ | 2.25  (1.32 - 3.81)^*^ | 2.18  (1.28 - 3.72)^*^ |
| No | 80 (1.9) | 62 (2.5) | 18 (1.1) |  |  |  |
| Yes | 4065 (98.1) | 2461 (97.5) | 1604 (98.9) |  |  |  |
|  |  |  |  |  |  |  |
| Disease Lists |  |  |  |  |  |  |
| Hypertension | 2469 (59.6) | 1524 (60.4) | 945 (58.3) | .18 | 0.92  (0.81 - 1.04 | 0.93  (0.82 - 1.06) |
| Diabetes mellitus | 1201 (29.0) | 729 (28.9) | 472 (29.1) | .88 | 1.01  (0.88 - 1.16) | 1.01  (0.88 - 1.17) |
| Bone disorders | 309 (7.5) | 153 (6.1) | 156 (9.6) | <.001**^*^** | 1.65  (1.31 - 2.08)**^*^** | 1.76  (1.39 - 2.24)**^*^** |
| Ophthalmic diseases | 145 (3.5) | 80 (3.2) | 65 (4.0) | .15 | 1.28  (0.91 - 1.78) | 1.22  (0.87 - 1.71) |
| Stroke | 1310 (31.6) | 755 (29.9) | 555 (34.2) | .004**^*^** | 1.22  (1.07 - 1.39)**^*^** | 1.20  (1.04 - 1.37)**^*^** |
| Coronary artery disease | 553 (13.3) | 328 (13.0) | 225 (13.9) | .42 | 1.08  (0.90 - 1.30) | 1.11  (0.92 - 1.34) |
| Arrhythmia | 103 (2.5) | 66 (2.6) | 37 (2.3) | .50 | 0.87  (0.58 - 1.31) | 0.88  (0.58 - 1.34) |
| Cancer | 106 (2.6) | 56 (2.2) | 50 (3.1) | .09 | 1.40  (0.95 - 2.06) | 1.34  (0.90 - 2.00) |
| Respiratory disorders | 530 (12.8) | 298 (11.8) | 232 (14.3) | .019**^*^** | 1.25  (1.04 - 1.50)**^*^** | 1.24  (1.03 - 1.50)**^*^** |
| Digestive disorders | 598 (14.4) | 379 (15.0) | 219 (13.5) | .18 | 0.88  (0.74 - 1.06) | 0.87  (0.72 - 1.04) |
| Urogenital disorders | 567 (13.7) | 326 (12.9) | 241 (14.9) | .08 | 1.18  (0.98 - 1.41) | 1.23  (1.02 - 1.48)**^*^** |
| Dementia | 1195 (28.8) | 752 (29.8) | 443 (27.3) | 0.09 | 0.89  (0.77 - 1.02) | 0.94  (0.81 - 1.08) |
| Psychiatric problems | 610 (14.7) | 366 (14.5) | 244 (15.1) | .63 | 1.04  (0.88 - 1.25) | 1.01  (0.84 - 1.20) |
| Mental retard | 92 (2.2) | 58 (2.3) | 34 (2.1) | .67 | 0.91  (0.59 - 1.40) | 0.90  (0.58 - 1.38) |
| Cerebral palsy | 31 (0.8) | 15 (0.6) | 16 (1.0) | .15 | 1.67  (0.82 - 3.38) | 1.72  (0.83 - 3.56) |
| Parkinsonism | 308 (7.4) | 189 (7.5) | 119 (7.3) | .86 | 0.98  (0.77 - 1.24) | 1.01  (0.80 - 1.29) |
| Spinal cord injury | 48 (1.2) | 14 (0.6) | 34 (2.1) | <.001**^*^** | 3.84  (2.05 - 7.18)**^*^** | 3.43  (1.82 - 6.46)**^*^** |
| Currently Infectious status | 21 (0.5) | 10 (0.4) | 11 (0.7) | .21 | 1.72  (0.73 - 4.05) | 1.75  (0.74 - 4.15) |
| Rare diseases | 11 (0.3) | 5 (0.2) | 6 (0.4) | .29 | 1.87  (0.57 - 6.14) | 1.79  (0.54 - 5.91) |
| Refractory epilepsy | 153 (3.7) | 90 (3.6) | 63 (3.9) | .59 | 1.09  (0.79 - 1.52) | 1.07  (0.76 - 1.50) |
| Others | 559 (13.5) | 320 (12.7) | 239 (14.7) | .06 | 1.19  (0.99 - 1.43) | 1.20  (1.00 - 1.45)  p = 0.0504 |

^a^aOR: adjusted for age, sex, and educational level

Supplemental Table 2 Association between ADL, IADL, Mood, Quality of Life and pain for those with dementia

| Covariates |  | Pain | | *p* value | Crude OR  (95% CI) | aOR^a^  (95% CI) |
| --- | --- | --- | --- | --- | --- | --- |
|  | All  (N = 4,145) | No  (N = 2,523) | Yes  (N = 1,622) |  |  |  |
| ADL^b^, mean ± SD | 20.4 ± 29.2 | 20.4 ± 29.7 | 20.3 ± 28.3 | .24 |  |  |
| ADL^b^ , *n* (%) |  |  |  | .18 |  |  |
| No dependence (100) |  | 56 (2.2) | 25 (1.5) |  | reference | reference |
| Dependence (0 - 99) |  |  |  |  | 1.45  (0.90 - 2.33) | 1.43  (0.88 - 2.31) |
| Mild (91 – 99) |  | 44 (1.7) | 18 (1.1) |  | 0.92  (0.45 - 1.89) | 0.88  (0.42 - 1.81) |
| Moderate (61- 90) |  | 243 (9.6) | 163 (10.1) |  | 1.50  (0.90 - 2.51) | 1.44  (0.86 - 2.41) |
| Severe (21 – 60) |  | 431 (17.1) | 300 (18.5) |  | 1.56  (0.95 - 2.56) | 1.50  (0.91 - 2.47) |
| Total (0 – 20) |  | 1749 (69.3) | 1116 (68.8) |  | 1.43  (0.89 - 2.30) | 1.42  (0.88 - 2.31) |
|  |  |  |  |  |  |  |
| IADL^c^, mean ± SD | 0.69 ± 1.31 | 0.66 ± 1.30 | 0.74 ± 1.31 | .002**^*^** |  |  |
| IADL^c^ , *n* (%) |  |  |  | .65 |  |  |
| No dependence (8) |  | 8 (0.3) | 4 (0.3) |  | reference | reference |
| Dependence (0 - 7) |  |  |  |  | 1.29  (0.39 - 4.28) | 1.31  (0.39 - 4.37) |
| Mild (6 – 7) |  | 32 (1.3) | 16 (1.0) |  | 1.00  (0.26 - 3.83) | 0.98  (0.25 - 3.74) |
| Moderate (3 – 5) |  | 196 (7.8) | 139 (8.6) |  | 1.42  (0.42 - 4.80) | 1.42  (0.42 - 4.81) |
| Severe (0 – 2) |  | 2287 (90.7) | 1463 (90.2) |  | 1.28  (0.39 - 4.26) | 1.30  (0.39 - 4.35) |
|  |  |  |  |  |  |  |
| Emotional/behavioral problems, *n* (%) |  |  |  |  |  |  |
| No |  | 1625 (64.4) | 970 (59.8) | .003**^*^** | reference | reference |
| Yes |  | 898 (35.6) | 652 (40.2) |  | 1.22  (1.07 - 1.38)**^*^** | 1.19  (1.05 - 1.36)**^*^** |
|  |  |  |  |  |  |  |
| Mean EQ5D scores, mean ± SD (health: 1, -1.0259 ~ 1) | -0.07 ± 0.43  (*n* = 4139) | 0.05 ± 0.36  (*n* = 2522) | -0.27 ± 0.46  (*n* = 1617) | <.001**^*^** |  |  |

^a^aOR: adjusted for age, sex, and educational level

^b^ADL means activities of daily living, and the total score for ADL ranges from 0 to 100

^c^IADL means instrumental activities of daily living, and the total score for IADL ranges from 0 to 8

Supplemental Table 3 Association between comorbidities and pain for those without dementia

| Covariates |  | Pain | | *p* value | Crude OR  (95% CI) | aOR^a^  (95% CI) |
| --- | --- | --- | --- | --- | --- | --- |
|  | All  (N = 616) | No  (N = 306) | Yes  (N = 310) |  |  |  |
|  |  | *n* (%) | *n* (%) |  |  |  |
| Comorbidities |  |  |  | <.001^*^ | 4.73  (1.77 - 12.65)^*^ | 4.93  (1.82 - 13.4)^*^ |
| No | 27 (4.4 | 22 (7.2) | 5 (1.6) |  |  |  |
| Yes | 589 (95.6) | 284 (92.8) | 305 (98.4) |  |  |  |
|  |  |  |  |  |  |  |
| Disease Lists |  |  |  |  |  |  |
| Hypertension | 341 (55.4) | 169 (55.2) | 172 (55.5) | .95 | 1.01  (0.74 - 1.39) | 0.94  (0.67 - 1.31) |
| Diabetes mellitus | 195 (31.7) | 96 (31.4) | 99 (31.9) | .88 | 1.03  (0.73 - 1.44) | 0.99  (0.70 - 1.41) |
| Bone disorders | 57 (9.3) | 29 (9.5) | 28 (9.0) | 0.85 | 0.95  (0.55 - 1.64) | 0.87  (0.50 - 1.52) |
| Ophthalmic diseases | 25 (4.1) | 8 (2.6) | 17 (5.5) | .07 | 2.16  (0.92 - 5.08) | 2.58  (1.04 - 6.41)^＊^ |
| Stroke | 149 (24.2) | 77 (25.2) | 72 (23.2) | .57 | 0.90  (0.62 - 1.30) | 1.02  (0.70 - 1.50) |
| Coronary artery disease | 85 (13.8) | 41 (13.4) | 44 (14.2) | .77 | 1.07  (0.68 - 1.69) | 0.95  (0.59 - 1.53) |
| Arrhythmia | 15 (2.4) | 5 (1.6) | 10 (3.2) | .20 | 2.01  (0.68 - 5.94) | 2.01  (0.67 - 6.04) |
| Cancer | 21 (3.4) | 11 (3.6) | 10 (3.2) | .80 | 0.89  (0.37 - 2.14) | 0.95  (0.39 - 2.29) |
| Respiratory disorders | 47 (7.6) | 20 (6.5) | 27 (8.7) | .31 | 1.36  (0.75 - 2.49) | 1.54  (0.83 - 2.86) |
| Digestive disorders | 84 (13.6) | 35 (11.4) | 49 (15.8) | .11 | 1.45  (0.91 - 2.32) | 1.33  (0.83 - 2.14) |
| Urogenital disorders | 77 (12.5) | 29 (9.5) | 48 (15.5) | .024**^*^** | 1.75  (1.07 - 2.86)**^*^** | 2.28  (1.36 - 3.82)**^*^** |
| Dementia | 6 (1.0) | 2 (0.7) | 4 (1.3) | .42 | 1.99  (0.36 - 10.9) | 1.76  (0.31 - 9.95) |
| Psychiatric problems | 87 (14.1) | 38 (12.4) | 49 (15.8) | .23 | 1.32  (0.84 - 2.09) | 1.20  (0.74 - 1.92) |
| Mental retard | 1 (0.2) | 0 | 1 (0.3) | 1.00 | - | - |
| Cerebral palsy | 5 (0.8) | 2 (0.7) | 3 (1.0) | 1.00 | 1.49  (0.25 - 8.95) | 1.71  (0.27 - 10.7) |
| Parkinsonism | 28 (4.6) | 15 (4.9) | 13 (4.2) | .67 | 0.85  (0.40 - 1.82) | 0.84  (0.39 - 1.83) |
| Spinal cord injury | 32 (5.2) | 12 (3.9) | 20 (6.5) | .16 | 1.69  (0.81 - 3.52) | 1.88  (0.88 - 4.00) |
| Currently Infectious status | 6 (1.0) | 1 (0.3) | 5 (1.6) | .22 | 5.00  (0.58 - 43.0) | 6.73  (0.78 - 58.5) |
| Rare diseases | 2 (0.3) | 0 | 2 (0.7) | .50 | - | - |
| Refractory epilepsy | 11 (1.8) | 4 (1.3) | 7 (2.3) | .55 | 1.74  (0.51 - 6.02) | 1.88  (0.52 - 6.83) |
| Others | 91 (14.8) | 38 (12.4) | 53 (17.1) | .10 | 1.45  (0.93 - 2.28) | 1.56  (0.99 - 2.48) |

^a^aOR: adjusted for age, sex, and educational level

Supplemental Table 4 Association between ADL, IADL, Mood, Quality of Life and pain for those without dementia

| Covariates |  | Pain | | *p* value | Crude OR  (95% CI) | aOR^a^  (95% CI) |
| --- | --- | --- | --- | --- | --- | --- |
|  | All  (N = 616) | No  (N = 306) | Yes  (N = 310) |  |  |  |
| ADL^b^, mean ± SD | 56.3 ± 32.3 | 58.9 ± 32.5 | 53.9 ± 31.9 | .04**^*^** |  |  |
| ADL^b^, *n* (%) |  |  |  | .13 |  |  |
| No dependence (100) |  | 50 (16.3) | 32 (10.3) |  | reference | reference |
| Dependence (0 - 99) |  |  |  |  | 1.70  (1.06 - 2.73)^＊^ | 1.55  (0.95 - 2.53) |
| Mild (91 – 99) |  | 18 (5.9) | 15 (4.8) |  | 1.30  (0.58 - 2.95) | 1.06  (0.46 - 2.46) |
| Moderate (61- 90) |  | 91 (29.7) | 88 (28.4) |  | 1.51  (0.89 - 2.57) | 1.41  (0.82 - 2.42) |
| Severe (21 – 60) |  | 97 (31.7) | 109 (35.2) |  | 1.76  (1.04 - 2.96)^＊^ | 1.64  (0.96 - 2.81) |
| Total (0 – 20) |  | 50 (16.3) | 66 (21.3) |  | 2.06  (1.16 - 3.67)^＊^ | 1.90  (1.04 - 3.45)^＊^ |
|  |  |  |  |  |  |  |
| IADL^c^, mean ± SD | 3.05 ± 2.15 | 3.22 ± 2.24 | 2.87 ± 2.04 | .07 |  |  |
| IADL^c^, *n* (%) |  |  |  | 05 |  |  |
| No dependence (8) |  | 19 (6.2) | 9 (2.9) |  | reference | reference |
| Dependence (0 - 7) |  |  |  |  | 2.21  (0.99 - 4.97) | 2.00  (0.87 - 4.56) |
| Mild (6 – 7) |  | 38 (12.4) | 25 (8.1) |  | 1.39  (0.54 - 3.56) | 1.27  (0.49 - 3.30) |
| Moderate (3 – 5) |  | 113 (36.9) | 126 (40.7) |  | 2.35  (1.02 - 5.41)^＊^ | 2.25  (0.96 - 5.25) |
| Severe (0 – 2) |  | 136 (44.4) | 150 (48.4) |  | 2.33  (1.02 - 5.32) | 2.03  (0.87 - 4.76) |
|  |  |  |  |  |  |  |
| Emotional/behavioral problems, *n* (%) |  |  |  |  |  |  |
| No |  | 19 (6.2) | 9 (2.9) | .049**^*^** | reference | reference |
| Yes |  | 287 (93.8) | 301 (97.1) |  | 1.22  (0.87 - 1.72) | 1.23  (0.87 - 1.74) |
|  |  |  |  |  |  |  |
| Mean EQ5D scores, mean ± SD (health: 1, -1.0259 ~ 1) | 0.23 ± 0.52  (*n* = 614) | 0.42 ± 0.46 | 0.05 ± 0.51  (*n* = 308) | <.001**^*^** |  |  |

^a^aOR: adjusted for age, sex, and educational level

^b^ADL means activities of daily living, and the total score for ADL ranges from 0 to 100

^c^IADL means instrumental activities of daily living, and the total score for IADL ranges from 0 to 8
